# The Enhanced Locating Performance of an Integrated Cross-Correlation and Genetic Algorithm for Radio Monitoring Systems

**DOI:** 10.3390/s140407541

**Published:** 2014-04-24

**Authors:** Yao-Tang Chang, Chi-Lin Wu, Hsu-Chih Cheng

**Affiliations:** 1 Department of Information Technology, Kao Yuan University, Kaohsiung 82151, Taiwan; E-Mail: t10066@cc.kyu.edu.tw; 2 Department of Electro-Optical Engineering, National Formosa University, Yunlin 632, Taiwan; E-Mail: thaliamuses@hotmail.com

**Keywords:** angle of arrival (AOA), time difference of arrival (TDOA), integrated cross-correlation and genetic algorithm (integrated CCGA), International Telecommunications Union Radiocommunication (ITU-R), Federal Communications Commission (FCC)

## Abstract

The rapid development of wireless broadband communication technology has affected the location accuracy of worldwide radio monitoring stations that employ time-difference-of-arrival (TDOA) location technology. In this study, TDOA-based location technology was implemented in Taiwan for the first time according to International Telecommunications Union Radiocommunication (ITU-R) recommendations regarding monitoring and location applications. To improve location accuracy, various scenarios, such as a three-dimensional environment (considering an unequal locating antenna configuration), were investigated. Subsequently, the proposed integrated cross-correlation and genetic algorithm was evaluated in the metropolitan area of Tainan. The results indicated that the location accuracy at a circular error probability of 50% was less than 60 m when a multipath effect was present in the area. Moreover, compared with hyperbolic algorithms that have been applied in conventional TDOA-based location systems, the proposed algorithm yielded 17-fold and 19-fold improvements in the mean difference when the location position of the interference station was favorable and unfavorable, respectively. Hence, the various forms of radio interference, such as low transmission power, burst and weak signals, and metropolitan interference, was proved to be easily identified, located, and removed.

## 1. Introduction

Radio frequency monitoring systems are applied worldwide to protect and maintain a favorable radio frequency environment that enables easy communication at any location and at any time using any device [1,2]. Since AOA monitoring/location stations cannot compensate for geographical changes that affect the radio frequency environment, the accurate performance of angle-of-arrival (AOA) location technology has degraded severely.

Wireless broadband communication technology has developed rapidly worldwide. Hence, the amount of the various forms of interference that can occur when using advanced communication technologies, such as low transmission power, burst and weak signals, and metropolitan interference, has increased rapidly too [3,4].

New monitoring and location technologies have been developed to prevent radio interference. Several previous studies have proposed integrating a time-difference-of-arrival (TDOA)-based location system with existing AOA-based location systems [5–9]. Hence, the complementary application of integrated TDOA/AOA location systems can improve the location accuracy and extend the location coverage area.

TDOA-based location technology involves two main processes, namely cross-correlation and solving hyperbolic algorithms [10]. General cross-correlation technology is beyond the scope of the current study, and solving hyperbolic algorithms is difficult because searching for the best solution resulting from a set of nonlinear equations is a crucial problem in TDOA location technology.

Fang and Chan have presented numerous methods for solving nonlinear hyperbolic algorithms [11,12] and have provided an exact closed-form solution that is not available when applying the Taylor-series method, which might converge to a local minimum when the initial input is unsatisfactory. In addition, a closed form solution for localization of distant transmitters was developed by using the triangulation of hyperbolic asymptotes rather than hyperbolae themselves [13]. To improve the location accuracy of previously proposed TDOA-based monitoring and location systems [10], a general cross-correlation and genetic algorithm (GA) [14–17] was proposed and evaluated in a metropolitan area, Tainan (Taiwan). The proposed integrated cross-correlation and GA (hereafter abbreviated as CCGA) was applied to identify the global optimal solution in nonlinear hyperbolic equations.

The remainder of this paper is organized as follows: Section 2 delineates the configuration of the TDOA-based monitoring and location system. Section 3 describes the experimental procedure of the proposed integrated CCGA. Subsequently, the simulation results obtained when applying the GA in extending the number of locations and in a three-dimensional (3D) environment are provided in Section 4. Furthermore, the experiments performed in Taiwan and the GA improvement are analyzed compared to the matrix calculation by computer in Section 5. Finally, Section 6 presents the conclusions.

## 2. Design of the Time-Difference-of-Arrival-Based Monitoring and Location System

In general, the following radio-spectrum monitoring technology is applied: (1) time of arrival (TOA), and (2) the AOA recommended in the 1995 and 2010 International Telecommunications Union Radiocommunication (ITU-R) spectrum monitoring handbooks, respectively [1,2]. In these publications, the monitoring site installation procedure and measuring equipment to execute the monitoring and location of radio interference stations is described. Furthermore, the advantages and disadvantages of TDOA-based technologies are presented in the ITU-R Report SM.2211 [18]. The transmitter and receiver must be precisely synchronized when using TOA location technology. The AOA of location technology is limited by non-line-of-sight (NLOS) and multipath effects. Hence, low transmission power, burst and weak signals, and metropolitan interference are difficult to identify and analyze (locate) using AOA technology. The proposed TDOA technology can be used to overcome the aforementioned shortfalls and is presented as follows.

### 2.1. Concept of the Time-Difference-of-Arrival-Based Location System

When applying the conventional TDOA scheme, two pairs of TDOA estimates are obtained using a cross-correlation algorithm [19–25]. The two pairs of estimates are then converted into two pairs of range (distance) difference measurements that in TDOA are multiplied by the propagation speed of electromagnetic waves (*i.e.*, 3 × 10^8^ m/s). The position (*X_T_*,*Y_T_*) is achieved by identifying the solutions to both Equations (1) and (2) because two hyperbolic curves are created to determine a crossing point (one TDOA estimate can be used to create one hyperbolic curve):

(1) $$d_{AB}=\sqrt{{\left(X_{T}-X_{A}\right)}^{2}+{\left(Y_{T}-Y_{A}\right)}^{2}-{\left(X_{T}-X_{B}\right)}^{2}+{\left(Y_{T}-Y_{B}\right)}^{2}}$$

(2) $$d_{AC}=\sqrt{{\left(X_{T}-X_{A}\right)}^{2}+{\left(Y_{T}-Y_{A}\right)}^{2}-{\left(X_{T}-X_{C}\right)}^{2}+{\left(Y_{T}-Y_{C}\right)}^{2}\;}$$

where (*X_A_*,*Y_A_*), (*X_B_*,*Y_B_*), and (*X_C_*,*Y_C_*) denote the known positions (*i.e.*, longitude and latitude) of three TDOA monitoring stations, and *d_ij_* denotes the difference in range (distance) between monitoring stations *i* and *j*. The coordinates (*X_A_*,*Y_A_*) are generally designed as the position of the central control monitoring station configured with the central control server, and (*X_T_*,*Y_T_*) denotes an unknown position of the measured transmitter (*i.e.*, interference signal).

TDOA-based location technology involves two main processes, namely cross-correlation and solving hyperbolic algorithms based on Equations (1) and (2) [10]. The cross-correlation algorithm calculates the time difference of arrival (TDOA) between a pair of TDOA-based stations. Subsequently, the TDOA is converted into the difference in range between the pair of TDOA-based stations, as shown in Equations (1) and (2).

In the first process, the general cross-correlation method is used to estimate the time delay coming from the interfering station and the emission signal of the interfering station is simultaneously detected between pairs of location stations. In the second process solving the hyperbolic algorithm presented in Equations (1) and (2) is difficult because searching for the best solution resulting from a set of nonlinear equations is a crucial problem in TDOA location technology.

In theory, the Equations (1) and (2) can only intersect at one point. In practice, since the effect of non-line of sight, multi-path and the presence of noise (e.g., the residual errors of GPS synchronization) results in the variance of a pair of hyperbolae (*i.e.*, the error of time difference of arrival), the true location of the emitter (*i.e.*, refer to as interference station) should be inside the region enclosed by the overlap of a pair of hyperbolae spreading results. In addition, ghost targets (*i.e.*, ambiguous problem) occur because a pair of symmetry hyperbolae is characterized by a leading and lagging time to intersect at two locations for a positive range distance.

The proposed radio spectrum monitoring system, which follows the ITU-R spectrum monitoring requirement [1,2] and installed in the current study, can provide additional information, including the radio frequency and power spectrum of the interference signal to identify various targets. Here, the higher power spectrum density denotes a shorter distance from the emitter (*i.e.*, interference station) to the TDOA monitoring station and then can distinguish either lag or lead time differences of arrival on hyperbolae.

Furthermore, in order to improve the location accuracy in current TDOA monitoring systems, selecting the arbitrary TDOA station #A or #B is installed with AOA configuration to provide additional bearing line information on TDOA complementary application [5,10,26]. Hence, the integrated TDOA/AOA monitoring system was applied to enhance the location accuracy by the current authors in previous work [10]. In the current experimental design, the AOA monitoring center can be installed in TDOA #1 (Luzhu, Kaohsiung Science Park presented in below section 3). That is, the TDOA #1 is characterized by TDOA and AOA measurements to enhance the location accuracy. Hence, the combination of TDOA #1 and an additional AOA technique at the control center station can process both hyperbolae (TDOA) and bearing line (AOA) calculations to significantly resolve the effect of ghost targets, non-line of sight and multi-path. Importantly, since the localization error of the spreading region enclosed by the spreading region of a pair of hyperbolae, the proposed genetic algorithm must be properly modified with selection, crossover, mutation procedures and the definition of fitness function to make an accurate convergence requirement in error tolerance.

### 2.2. Configuration of the Proposed Time-Delay-of-Arrival-Based Monitoring and Location System

**Figure 1.**
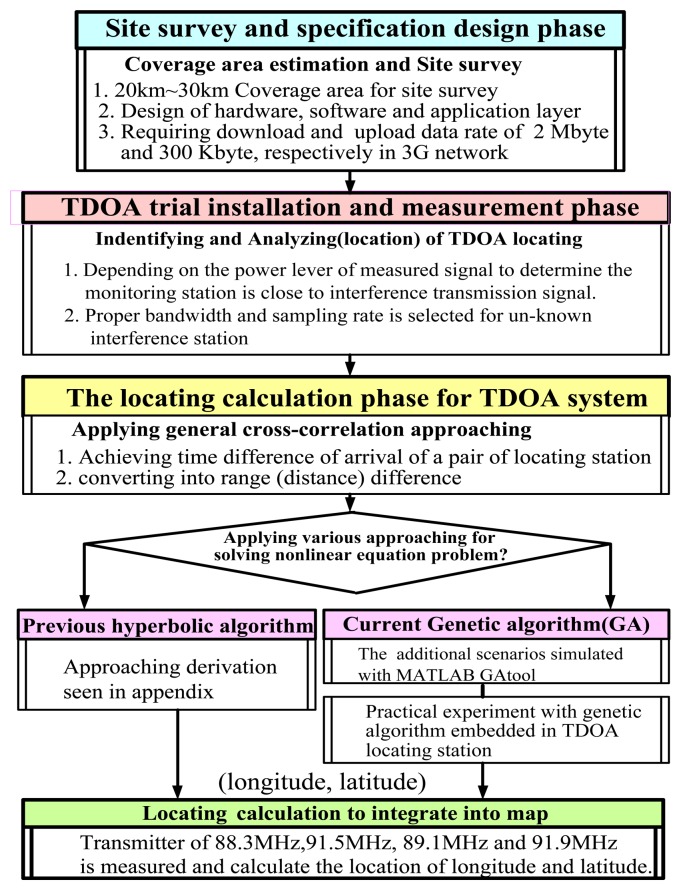
The major installation processing consist of below step in current proposed TDOA-based monitoring station.

As shown in Figure 1, the configuration process consists of:

(1) a site survey and specification design phase;
(2) a TDOA trial installation and measurement phase;
(3) a phase in which the location is calculated using a cross-correlation approach;
(4) a phase in which a hyperbolic equation is solved; and
(5) a phase in which the position of the interference transmitter (*X_T_*,*Y_T_*) is added to a geographical map.

The design configuration the TDOA monitoring and location system [11,12] consists of hardware, a cross-correlation and solving hyperbolic algorithm, and a user interface. Importantly, an evaluation criterion is applied to verify the performance of the location accuracy. When TDOA is applied, multiple location stations are used to measure the time required for a pending signal to arrive at each location station receiver.

**Figure 2.**
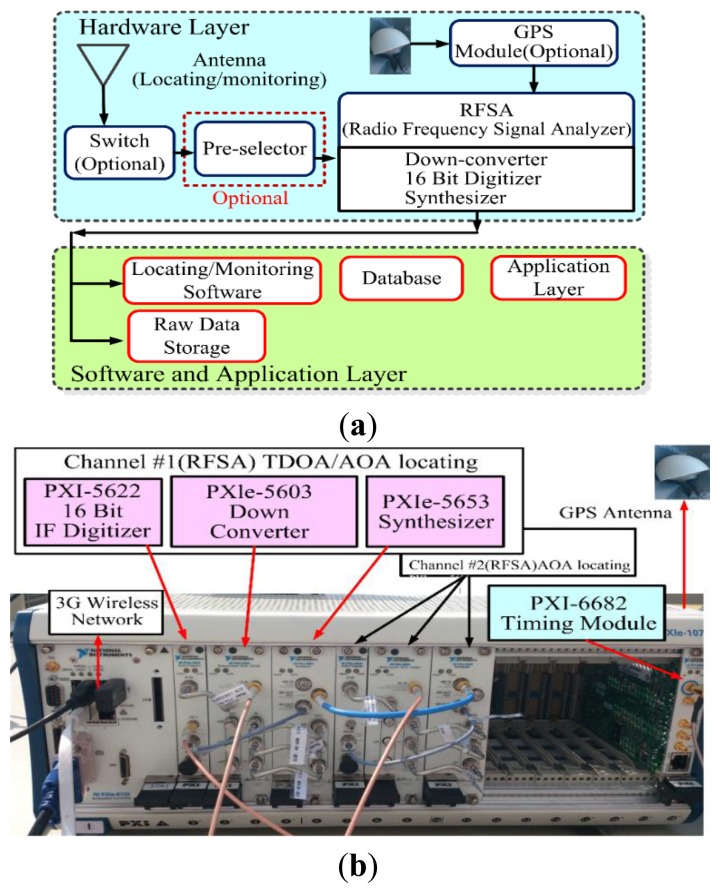
The proposed TDOA monitoring/locating system (**a**) system architecture (**b**) physical configuration in current experiments.

The hardware and software of the TDOA technology were designed based on the performance of the receiver, time synchronization, and network connectivity observed in the experiments to ensure that accurate solutions were obtained. The architecture of the monitoring and location system and the location requirements are shown in Figure 2.

Signal synchronization is crucial in TDOA-based monitoring. TDOA-based monitoring and location systems typically use a GPS receiver to establish a common time reference. For the various TDOA monitoring station located in different geographical area, the residual errors of GPS synchronization results from various satellite clocks and the transmission environment, such as of ionosphere delay and tropospheric delay, *etc*.

In order to connect together each monitoring station for time synchronization, a digital circuit switch network (e.g., PDH, SDH network and fixed line) is better than a packet switch network (*i.e.*, IP network) for precise synchronicity accuracy. Here, the leased line (fixed fiber-to-the-building, FTTB) for the active master line and broadband integrated service digital network (B-ISDN) for the back-up line are employed, respectively, in the current experiment.

Furthermore, many synchronization polices are designed to compensate different link delays, including NTP, RBS, TPSN, FTSP, GTSP with time synchronization and the Whistle method without time synchronization, respectively [26–31]. For the commercial application of the radio spectrum monitoring system installed in the current study, the acquired time stamp signal from a GPS antenna is transported by the master monitoring station and used in slave monitoring (TDOA #2 and #3 presented in below section 3) to steer the local clocks. Here, the master monitoring station (*i.e.*, TDOA #1) is installed in Luzhu, Kaohsiung Science Park (*i.e.*, called central control station server) and two slave monitoring stations are installed in TDOA # 2 and #3, respectively, as seen in below section 3.

Like previous configurations [10], the experimental monitoring station used in this study was configured in three steps, namely hardware selection, software design (cross-correlation and solving hyperbolic equations algorithm), and estimation of location errors.

To ensure that the location performance is favorable, TDOA technology depends on the precise identification and estimation of frequency, bandwidth, and modulation mode coming from unknown emission stations to determine the optimum sample rate of received signals. In this experiment, frequency modulation (FM) characterized by a 200 KHz bandwidth is assumed as the interference station. The FM transmission signal was detected for a 1-min time interval to execute the TDOA location processing.

The sample was optimized to improve the cross-correlation peak by selecting the proper bandwidth of the filter according to the difference in the arrival time. Unlike AOA technology, no demodulation is conducted in the sampling process when using TDOA technology. The transmission signal in the time domain is combined with the GPS time stamp to execute the cross-correlation algorithm at the central station.

**Figure 3.**
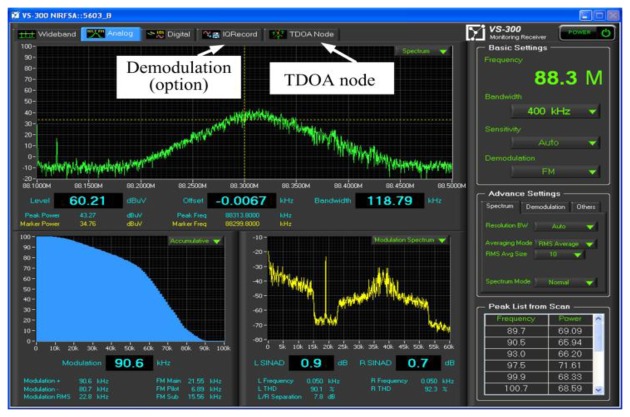
The proposed interface of the TDOA-based monitoring/locating system by selecting the sampling time, filter, bandwidth, *etc*.

According to the ITU-R spectrum monitoring handbook 1995, the TDOA monitoring and location system must feature the monitoring function, thus enabling the operator to easily identify the variation in specific interference signals for a modulation type and, subsequently, select a precise sampling rate at each TDOA-based monitoring station. Hence, the user interface was designed as shown in Figure 3.

**Figure 4.**
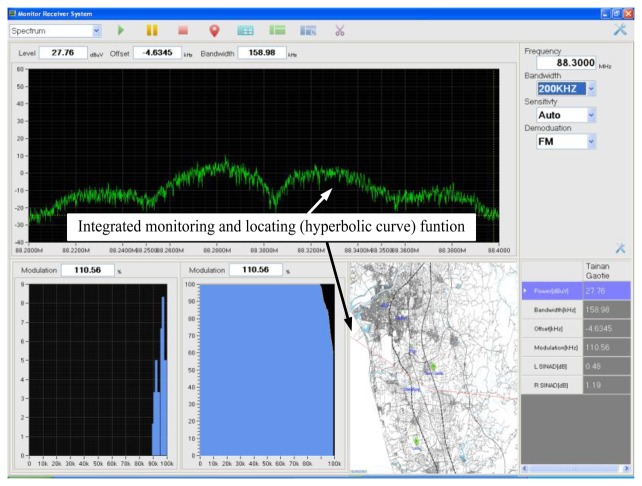
The proposed user interface on integrating the monitoring power spectrum density and locating position by indicating hyperbolic curve.

Three TDOA-based monitoring stations were installed in this study. Therefore, three pairs of TDOA estimates and three pairs of hyperbolic curves were obtained. To enhance the location accuracy of the proposed TDOA monitoring and location system, the user interface must be designed such that the hyperbolic curve exhibiting a favorable location performance can be observed in the operating screen. Hence, the operator is easy to select better location line from calculated hyperbolic curve, as shown in the lower right corner of Figure 4.

## 3. Experimental Design Conducted to Test the Time-Difference-of-Arrival-Based Monitoring and Location System

**Figure 5.**
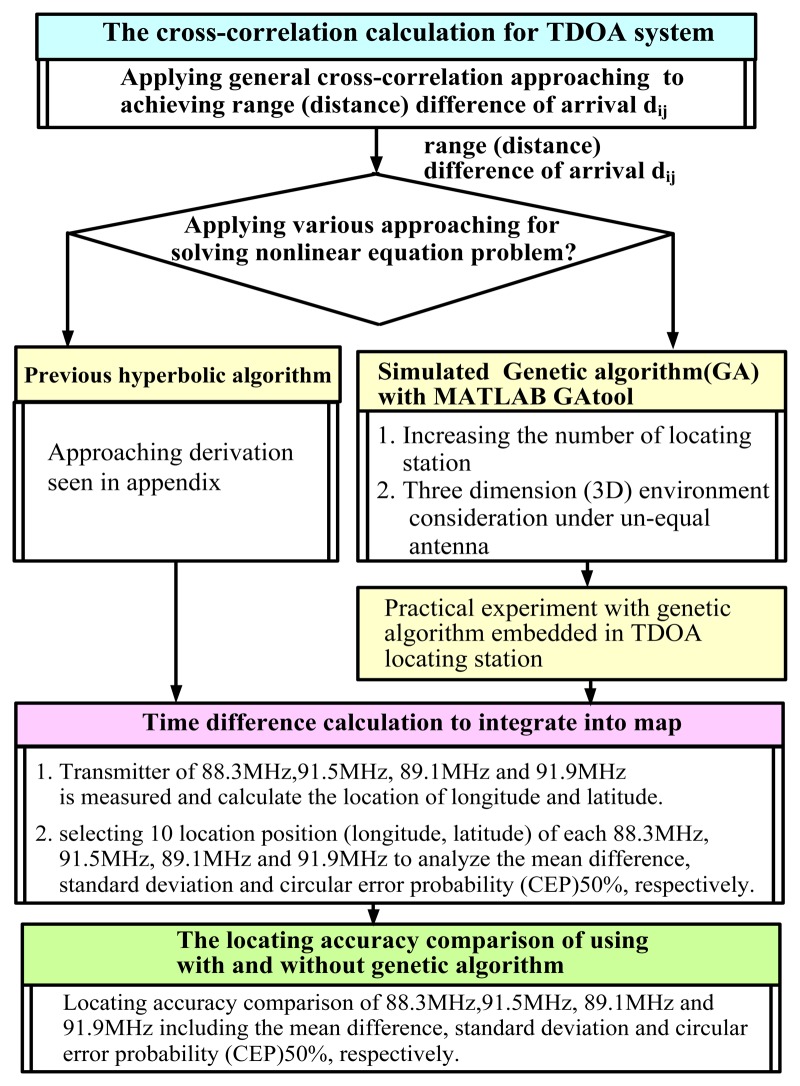
The experimental processing of proposed and conventional TDOA-based monitoring/location system.

Based on the process shown in Figure 1, the experimental design was developed further, as shown in Figure 5. TDOA-based location technology involves two major processes, namely cross-correlation and solving hyperbolic algorithms. The cross-correlation approach entails calculating the TDOA based on a pair of TDOA-based location stations. General cross-correlation technology is beyond the scope of this study.

Solving hyperbolic algorithms is difficult because solving a set of nonlinear equations presented in Equations (1) and (2) is the biggest challenge to find the best solution in TDOA location accuracy [11,12]. Except for the location solution obtained by the approach of solving nonlinear hyperbolic equations, a novel closed-form location estimator was developed for TDOA emitter localization based on triangulation of the TDOA hyperbolic asymptotes rather than the TDOA hyperbolae themselves applied in current study. By employing the clustering the bearing angles of the linear asymptotes with a kernel density estimate, the solution of associating the linear asymptotes with the emitter is presented to resolve significant location errors and ghost targets arising from the presence of moderate noise without the consideration of multipath distortion, multiple and moving interference emission [13].

**Figure 6.**
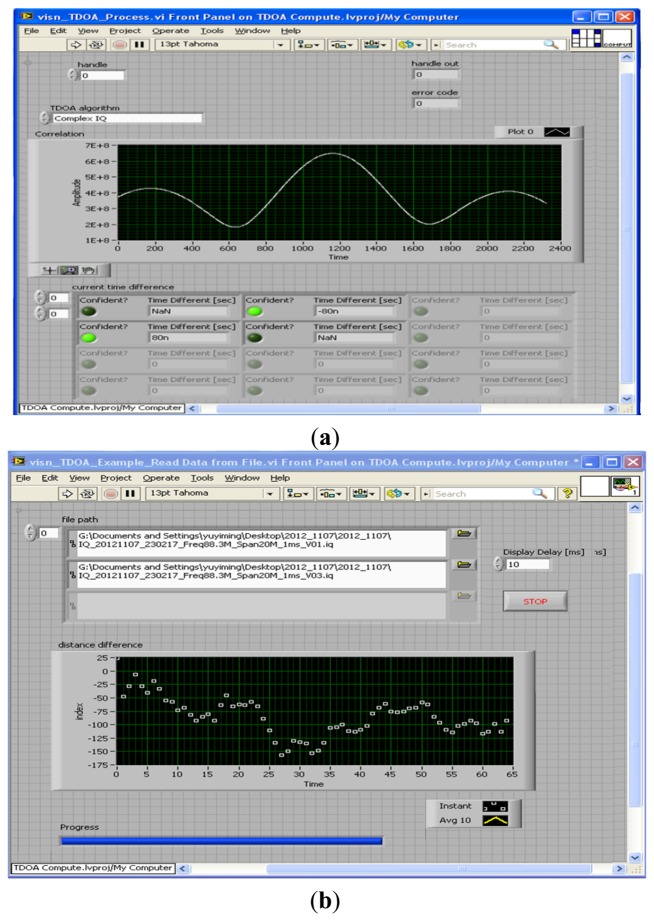
The 88.3 MHz experimental evaluation of (**a**) time difference and (**b**) distance difference using cross-correlation algorithm in TDOA-based monitoring/locating system.

By using the general cross-correlation algorithm shown in Figure 6, an example of the time difference at 88.3 MHz was obtained. Subsequently, the range (distance) difference was obtained that the time difference of arrival is multiplied by the speed of radio waves (3 × 10^8^ m/s).

In this study, two types of algorithm for solving hyperbolic equations were evaluated, as shown in Figure 5. One approach involved solving the hyperbolic equations by using the derivation process described in the Appendix. The second approach involved applying the GA in a practical experiment.

First, to improve the location accuracy, various scenarios, such as an extended TDOA-based location station (similar to sensor networks) and a 3D environment (considering an unequal location antenna configuration) were investigated.

**Figure 7.**
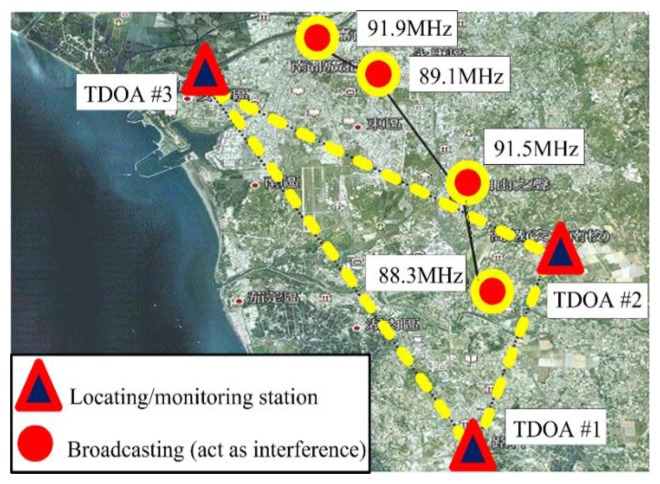
The proposed experiment configuration for investigating the TDOA limitation of location performance in multi-path effect of metropolitan area.

Second, the proposed CCGA was evaluated in the metropolitan area of Tainan. Finally, according to the ITU-R Report SM.2211 [18], the location performance of TDOA-based technology depends strongly on the position of the measured transmitter station. Figure 7 illustrates favorable and unfavorable performance at the central point of the triangle and away from the TDOA-based coverage area, respectively. The most unfavorable scenario in the experiment occurred when the measured station broadcasted at 91.9 MHz.

In the trial experiment, three monitoring stations, including the Jinkang and Gaote stations in the Tainan metropolitan area (*i.e.*, southern Taiwan), were selected to create an appropriate triangular arrangement. The central station was established at Luzhu (Kaohsiung Science Park). A distance of approximately 9.94 km was calculated between Jinkang station and Luzhu. As shown in Figure 7, the proposed experimental configuration is major implemented in the metropolitan area. Hence, it is easy to prove the TDOA location reliability and accuracy even the decay of the location performance resulting from the multi-path effect [3,4,18–25].

The central station, AOA/TDOA #1, was installed in Luzhu. TDOA #2 and TDOA #3 (Tainan metropolitan area) acted as remote monitoring stations. These monitoring stations were selected by considering the effects of the barriers created by buildings, ground reflection, and reflected waves. In addition, an upload transmission rate suitable for a 3G mobile network was required. The positions (longitude and latitude) of the TDOA-based monitoring and transmission stations are listed in Table 1.

Each specific central frequency (*i.e.*, 88.3 MHz, 91.5 MHz, 89.1 MHz, and 91.9 MHz) was recorded for 200 s in a 3–4-Mbyte storage file, and the monitoring transmission signal of the receiver was then played back to calculate the location error.

The three monitoring signals were simultaneously transferred to the central control station via the remote stations, and the time difference of the three pairs of measured signals was then obtained using the integrated CCGA.

## 4. Enhancing the Accuracy of the Time-Difference-of-Arrival-Based Location System Using the Genetic Algorithm in Simulations

TDOA estimation is divided into two stages. The first stage involves estimating the time delay to determine the TDOA between pairs of location station. In the second stage, the TDOA value is converted into a value of pairs of range (distance) difference, and a set of nonlinear hyperbolic equations is obtained. Available algorithms are then used to determine the exact solution of the nonlinear hyperbolic equations in the second stage. Because solving multiple equations involving highly nonlinear problems is difficult, several iterative methods, including the maximum likelihood estimation and constrained optimization methods, have been proposed.

Location algorithms are generally categorized as deterministic or stochastic computing algorithms. Deterministic algorithms, such as the aforementioned algorithms proposed by Fang and Chan, as well as Taylor series expansion [11,12], are applied when using direct or iterative methods.

The approach of Fang is characterized by favorable location performance in LOS scenarios, but not in the locating station scenario. However, when the location error at a locating station is substantial, the performance of the algorithm proposed by Fang is severely affected.

The algorithm proposed by Chan features low computational complexity and favorable location performance when a propagation channel model with additive white Gaussian noise (AWGN) is used. However, the location performance rapidly declines in non-LOS scenarios.

When using the Taylor series expansion algorithm, the initial position for iterative processing must be estimated. By obtaining the local minimum square solution, the estimated position is improved in each iterative process and the computational complexity increases considerably. The Taylor series expansion algorithm can be adapted to various types of propagation in open-air environments. However, determining the initial position of an unknown station influenced by interference is difficult, thus reducing the location accuracy.

Stochastic computing algorithms are employed to identify the optimal solution within a matching condition pool. The termination condition is defined and assigned to prevent divergence or premature convergence toward local suboptimal results. Hence, the results generated by stochastic computing algorithms are characterized by slight differences in each iterative process, and these algorithms are suitable for solving searching space problems involving numerous data.

### 4.1. Features of the Genetic Algorithm Applied in Time-Difference-of-Arrival-Based Location Systems

The GA, which is classified as a stochastic computing algorithm, is an effective search method that has been used widely in identifying the optimal solutions of various problems. The GA mimics the mechanisms of natural evolution, in which stronger individuals are more likely to survive in a competitive environment. A GA treats each potential solution of a problem as an individual, represented by a string of parameter values in a binary or real form. These parameter values represent the genes of a chromosome. In GA evolution, the suitability of each chromosome as a solution to the optimization problem is indicated by its fitness value based on a specific objective fitness function. The GA commences by establishing a population pool of randomly selected chromosomes.

When inconsistencies exist among individuals (*i.e.*, the genes of a chromosome), a new generation of genes is created by employing the selection, crossover, and mutation processes. The fitness value of the new generation is evaluated, and evolution continues until the optimal solution or the termination condition is reached. The first generation is called the parent generation. The subsequent generation consists of the child chromosomes, which are created through crossover and mutation processes. The population consists of all generations.

Compared with iterative deterministic methods for identifying solutions such as the GA [14–17], direct solving methods, such as those of Fang and Chan and the Taylor series expansion algorithm [11,12], enable the global solution to be identified easily, and the solutions are not divergent or converge toward a local suboptimal result. However, when the transmission channel passes through an unfavorable open-air environment and the target is a mobile transmitter, using direct solving methods reduces the location accuracy and increases the complexity of calculation.

**Figure 8.**
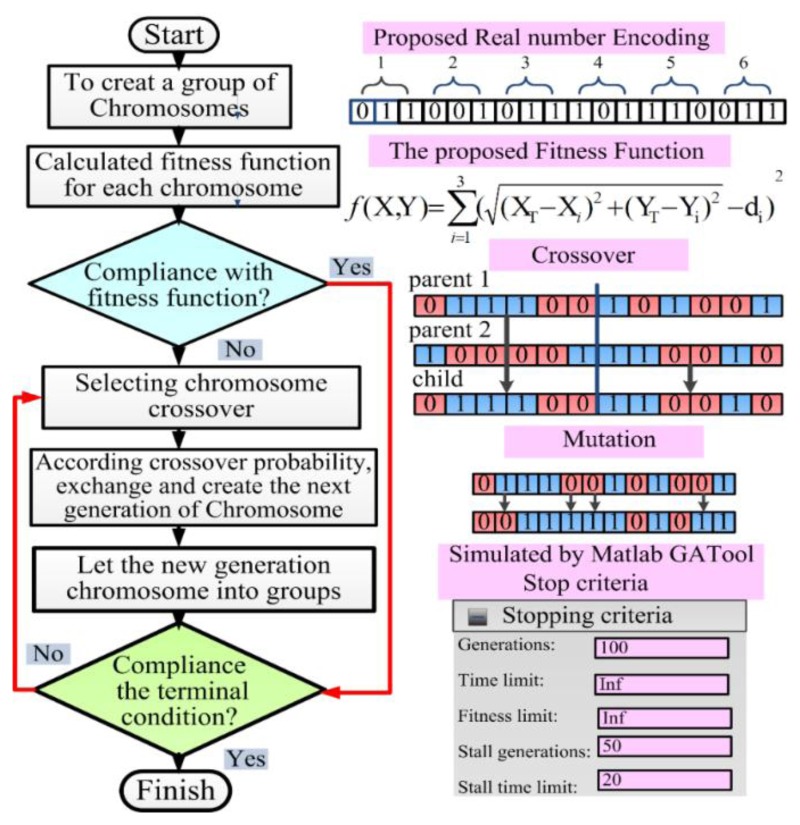
The procedure of proposed genetic algorithm applied in TDOA-based locating system.

In this study, the proposed GA featured parallel search ability and exhibited a high probability to prevent local suboptimal results, thus enabling the nonlinear hyperbolic equations in Equations (1) and (2) and the Appendix to be solved. In addition, the capability of the proposed GA to achieve the optimal global solution depends on the crossover and mutation processes as well as flexible terminal condition selection. The procedure used to apply the proposed GA in the TDOA-based location system is shown in Figure 8. The basic operations executed in the proposed GA approach are described briefly as follows:

(a) The GA commences by establishing an initial population pool of randomly selected chromosomes. As shown in Figure 8, the real genetic algorithm is applied to create the solutions of the parameters as a string of real values. Thus, a GA treats each potential solution of a problem as an individual, represented by a string of parameter values in a real form.
(b) In the recursive processing of the GA, the fitness value indicates the suitability of each chromosome. In other words, the fitness value is determined based on an objective fitness function and serves as a solution to the optimization problem. The fitness function for the location accuracy of three TDOA-based location systems is expressed as:
  (3) $$f(X,Y)=\sum_{i=1}^{3}{\left(\sqrt{{\left(X_{T}-X_{i}\right)}^{2}+{\left(Y_{T}-Y_{i}\right)}^{2}}-d_{i}\right)}^{2}$$
  where *i* denotes one of the TDOA-based monitoring stations; (*X_i_*,*Y_i_*) denotes the position (*i.e.*, coordinate values of longitude and latitude) of the *i*-th TDOA-based monitoring station; and *d_i_* is the distance between the *i*-th TDOA-based monitoring station and the interference transmitter (*X_T_*,X*_T_*) used to calculate the location error. The goal of the proposed GA scheme is to identify the optimal (*X*,*Y*) values, which are the values at which the fitness function *f* (*X*,*Y*) is minimal. Hence, (*X_T_*,*X_T_*) is the optimal solution for the coordinate values of the interference transmitter.
(c) In each recursive generation of the GA, the selection operator determines the individual strings that are copied into the mating pool and used as the basis for creating the subsequent generation. In general, a particular string characterized by a high probability that the value of the fitness function is minimal is copied into the mating pool. In this study, the most common method, roulette-wheel selection, was employed to select the candidate strings to be reproduced in a statistical fashion based only on their relative fitness function values.
(d) Crossover is an approach to moving through the space of possible solutions based on the information associated with existing solutions. In the crossover process, the GA selects two strings randomly from the mating pool and then determines whether or not crossover should occur by applying a parameter known as the crossover probability, *p*. In this study, the uniform crossover probability of *p* = 50% was employed.
(e) Mutation prevents a population of chromosomes from becoming too similar to each other, thereby slowing or stopping evolution, or causing the GA to converge toward a local suboptimal solution. In this study, uniform mutation was employed.

### 4.2. Improving the Performance of the Time-Difference-of-Arrival-Based Location System by Using the Genetic Algorithm

First, to simplify the development procedure, the GA tool of the MATLAB software application was employed to replace the hyperbolic algorithm shown in the Appendix. Second, the programming language created using the MATLAB software application was embedded into the digital signal-processing module of the TDOA-based location system configured on the central control station.

ITU-R Report SM.2211 [18] indicated that the various location performance is relative to the position of the emission station (*i.e.*, interference station). In this publication for qualitative analysis, the location accuracy of the central point of the triangle is better than that of away from the geographical coverage area in TDOA-based location station. In addition, the TDOA location performance relies on the topology and coverage area of location station and the interference type resulting from the low transmission power, burst/weak signals, and metropolitan emission.

**Figure 9.**
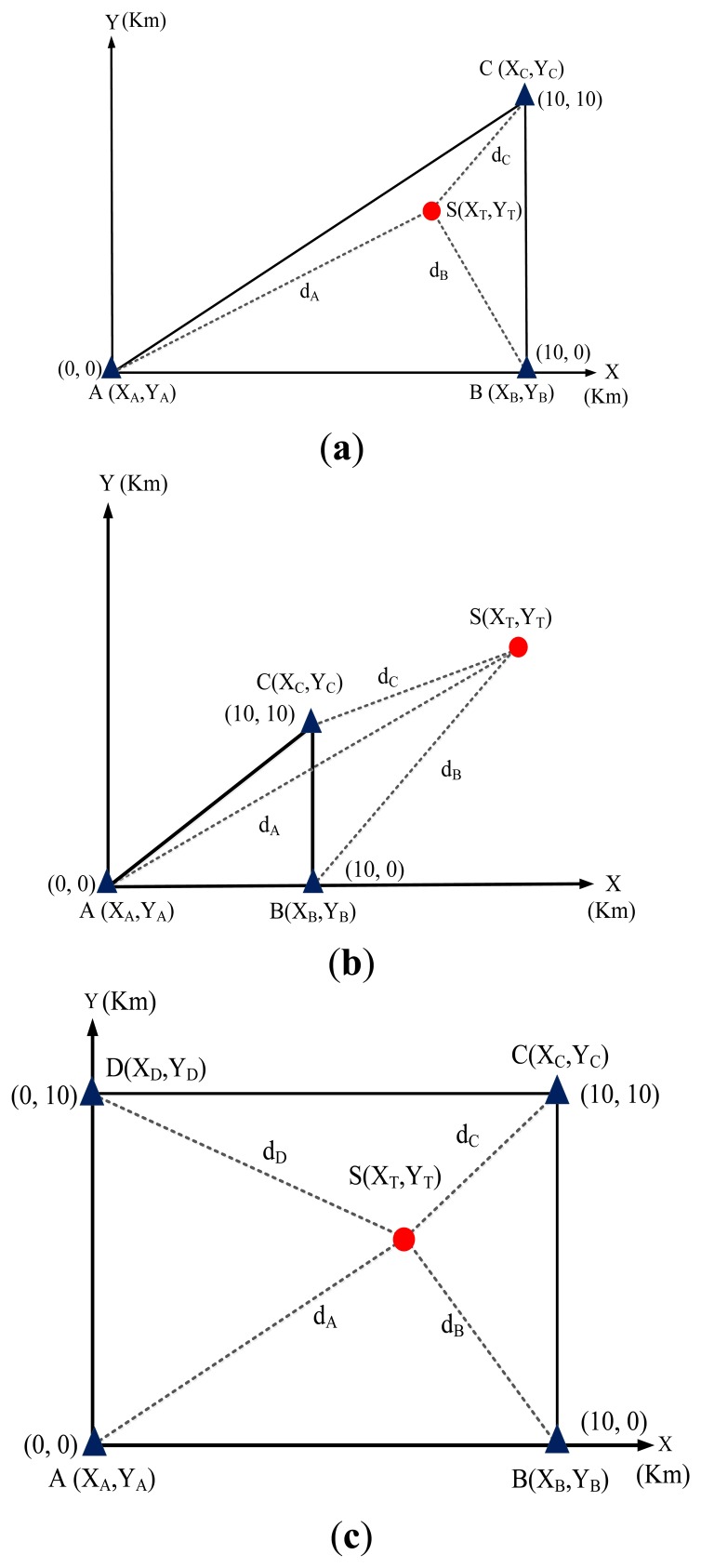
The simulated configuration of various scenarios on (**a**) inside coverage area (**b**) outside coverage area (**c**) spreading (extending) locating networks of TDOA-based locating system.

In the simulation, which was conducted by using the GA tool of the MATLAB software application, the signal of the transmitter station could be received from any direction, and the effects of power loss and diffraction in the transmission channel were neglected. Figure 9a–c show that the simulation configuration was varied to investigate the location accuracy in various scenarios (a) inside the coverage area of the TDOA-based location system and (b) outside the coverage area of TDOA-based location system. Furthermore, the location accuracy of spreading (extending) location networks (*i.e.*, sensor networks) was analyzed while the number of TDOA-based location systems was increased.

The antenna height of the three TDOA-based location stations was configured based on the same sea-level surface. The positions of the location stations were assigned to simulated coordinates of (0,0), (10,0), (0,10), and (10,10). The kilometer was the unit of distance.

**Figure 10.**
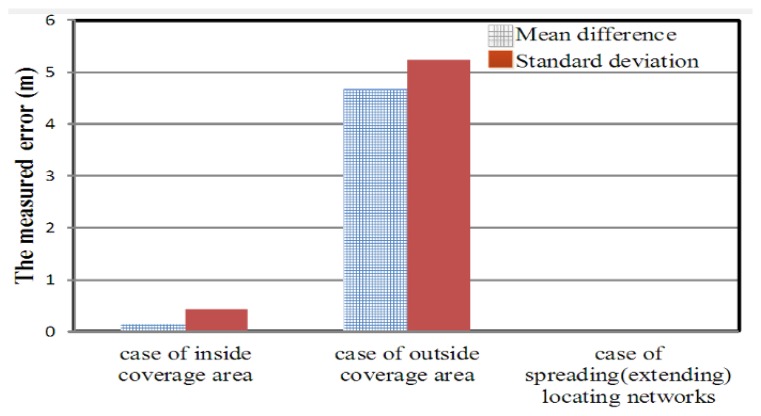
The locating accuracy by using proposed GA in the various scenarios presented in Figure 9.

In the simulation of the GA, the radiowave transmission speed was 3 × 10^8^ m/s (*i.e.*, electro-magnetic wave speed), the termination condition was 500 iterations, and obtaining the 10 groups (*X_T_*,*X_T_*) of location positions in the simulated processing. The results of simulating the proposed GA are shown in Figure 10. The GA exhibited more favorable performance than did the computing approaches presented in the Appendix.

In a practical scenario, the antennas of three TDOA-based location stations were installed on different buildings. The heights were varied to determine their effects on the location accuracy. To improve the location performance, the 3D location error was investigated.

**Figure 11.**
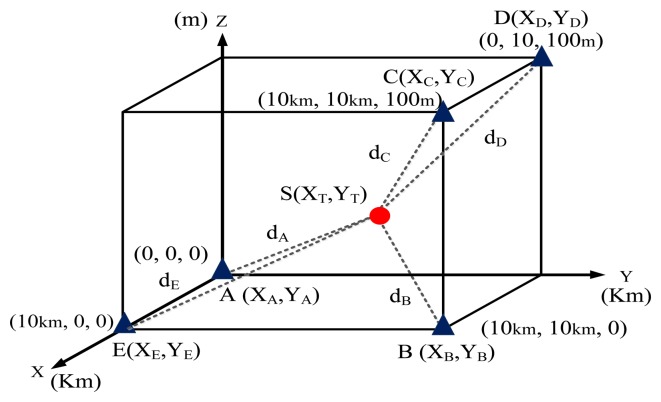
The 3D locating error analysis of monitoring/locating station (sensor station).

Figure 11 shows that the monitoring site was deployed in 3D free space. Five monitoring positions, Stations A, B, C, D, and E, were positioned at (0 km, 0 km, 0 m), (10 km,10 km,100 m), (10 km,10 km, 100 m), (0 km,10 km,100 m), and (10 km,0 km,0 m), respectively. The distance along the X and Y axes was measured in kilometers. The distance along the Z axis (the antenna height above sea-level surface) was measured in meters.

**Figure 12.**
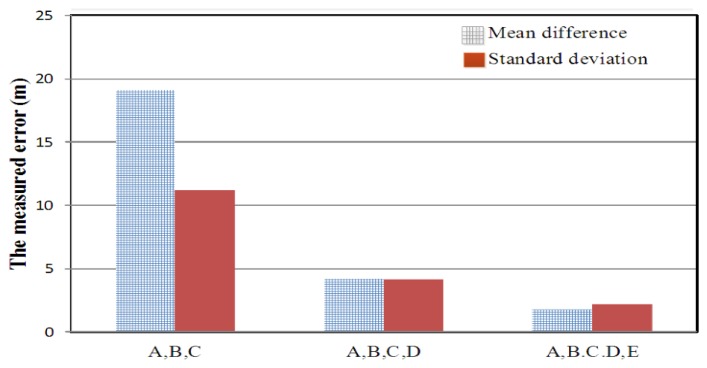
The locating accuracy by using the proposed genetic algorithm in a 3D free space environment.

As shown in Figure 12, the location error was analyzed while the number of monitoring sites was gradually increased.

## 5. Implementation of the Proposed Integrated CCGA Algorithm in a Time-Difference-of-Arrival-Based Location System

The location accuracy of the calculation approach presented in the Appendix and the GA were compared, as shown in Figure 5, and the 10 results measured at the 88.3-MHz transmitter (assumed interference station) when the GA was not used are shown in Table 2.

To enhance the accuracy of a previous experiment described in [10], the proposed integrated CCGA was implemented in the current study. The location performance of the proposed algorithm measured at an 88.3-MHz broadcasting station is shown in Table 3.

The experimental results regarding the performance of the hyperbolic algorithm and proposed GA in TDOA monitoring are summarized in Table 4. The location errors varied according to the position of the broadcasting station transmitting at a frequency of 88.3, 91.5, 89.1, and 91.9 MHz, respectively.

Table 4 indicates that favorable and unfavorable performance occurred at the central point of the triangle and outside the TDOA-based coverage area, respectively. The least favorable scenario occurred when the measured station broadcasted at 91.9 MHz.

The results indicated the worst location accuracy of 91.9 MHz at a circular error probability (CEP) of 50% was less than 60 m when the multipath effect was present. Compared with the results obtained when applying the hyperbolic algorithm in the conventional TDOA-based location system, the mean difference improved 17-fold (*i.e.*, 230.31/13.05) and 19-fold (*i.e.*, 929.26/48.87) at 88.3 MHz and 91.9 MHz, respectively. Moreover, the standard deviation improved 3-fold (*i.e.*, 22.24/6.49) and 11-fold (*i.e.*, 275.76/24.09) at 88.3 MHz and 91.9 MHz, respectively. Hence, proposed integrated CCGA was proved to exhibited high location accuracy, despite metropolitan interference.

## 6. Conclusions

The rapid development of wireless broadband communication technology has affected the location accuracy of worldwide radio monitoring stations that employ TDOA location technology, and the stations in Taiwan are no exception. In this study, TDOA-based location technology was implemented in Taiwan for the first time according to International Telecommunications Union Recommendation requirements regarding monitoring and location applications. To improve location accuracy, various scenarios, such as a three-dimensional environment (considering an unequal locating antenna configuration), were investigated.

Moreover, the proposed GA was integrated with a cross-correlation algorithm (CCGA) in a practical experiments for the first time. The results indicated that the location accuracy at a CEP of 50% was less than 60 m when a multipath effect was present in a metropolitan area.

Compared with results obtained when applying the hyperbolic algorithm in the conventional TDOA-based location system, the mean difference improved 17-fold and 19-fold at 88.3 MHz and 91.9 MHz, respectively. Moreover, the standard deviation improved 3-fold and 11-fold at 88.3 MHz and 91.9 MHz, respectively. Hence, the proposed CCGA scheme was proved to identify, locate, and remove radio interference resulting from low transmission power, burst and weak signals, or metropolitan.

In future work, the proposed integrated cross-correlation and genetic algorithm (CCGA) will be used to further investigate the location error resulting from more mobile detected target, and various environmental issues in the effect of non-line of sight (NLOS) and multi-path.

## Figures and Tables

**Figure A1.**
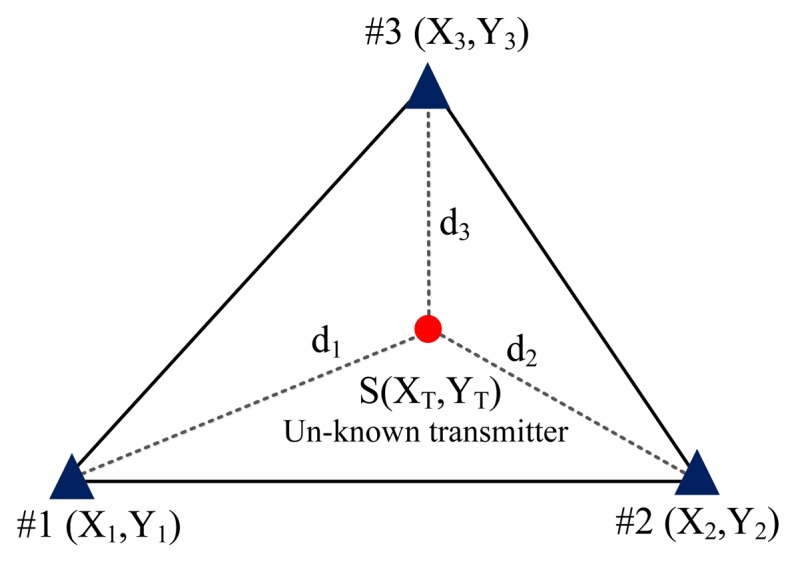
The demonstrated diagram of solving the location by using the previous computing algorithm.

**Table 1.** The proposed monitoring and transmission position of the proposed experiment.

**Proposed monitoring and measured broadcasting station**	**Longitude**	**Latitude**
TDOA #1 monitoring (Luzhu) Central control station	E 120°15′ 42.41″	N 22°50′ 21.70″
TDOA #2 monitoring station (Gaote)	E 120°17′ 9.00″	N 22°55′ 29.00″
TDOA #3 monitoring station (Jinkang)	E 120°10′ 14.60″	N 22°59′ 49.70″
88.3 MHz broadcasting station	E 120°16′ 26.36″	N 22°54′ 15.15″
91.5 MHz broadcasting station	E 120°15′ 29.00″	N 22°56′ 55.00″
89.1 MHz broadcasting station	E 120°13′ 45.00″	N 22°59′ 57.00″
91.9 MHz broadcasting station	E 120°12′ 33.00″	N 23°0′ 45.00″

**Table 2.** The location performance using without genetic algorithm for the measured 88.3 MHz broadcasting station.

**The measured broadcasting station of 88.3 MHz @Actual location (E 120.2656,N 22.9064)**						
**Location result index**	**Longitude**	**Range Error (m)**	**Maximum Range Error (m)**	**Minimum Range Error (m)**	**Mean Difference (m)**	**Standard Deviation (m)**
	**Latitude**					
1	E 120.2673	222.62	274.53	200.82	230.31	22.24
	N 22.9076					
2	E 120.2673	236.58				
	N 22.9078					
3	E 120.2671	200.82				
	N 22.9075					
4	E 120.2671	206.87				
	N 22.9076					
5	E 120.2673	251.54				
	N 22.9080					
6	E 120.2672	274.53				
	N 22.9076					
7	E 120.2672	214.47				
	N 22.9075					
8	E 120.2672	244.51				
	N 22.9080					
9	E 120.2672	221.55				
	N 22.9077					
10	E 120.2673	229.64				
	N 22.9077					

**Table 3.** The location performance using the proposed genetic algorithm for the measured 88.3 MHz broadcasting station.

**The measured broadcasting station of 88.3 MHz @Actual location (E 120.2656,N 22.9064)**						
**Location result index**	**Longitude**	**Range Error (m)**	**Maximum Range Error (m)**	**Minimum Range Error (m)**	**Mean Difference (m)**	**Standard Deviation (m)**
	**Latitude**					
1	E 120.2656	10.94	24.72	2.11	13.05	6.49
	N 22.9065					
2	E 120.2654	23.01				
	N 22.9064					
3	E 120.2656	10.37				
	N 22.9065					
4	E 120.2655	13.48				
	N 22.9065					
5	E 120.2656	15.89				
	N 22.9065					
6	E 120.2657	24.72				
	N 22.9062					
7	E 120.2656	6.95				
	N 22.9064					
8	E 120.2657	13.08				
	N 22.9064					
9	E 120.2656	9.95				
	N 22.9065					
10	E 120.2656	2.11				
	N 22.9064					

**Table 4.** The summarized results of the experiment for TDOA–based measured locations.

**Location Technology**	**Frequency (MHz)**	**Distributed Range (m)**	**Location accuracy @ CEP 50% (m)**	**Mean difference (m)**	**Standard deviation (m)**
Conventional Hyperbolic algorithm for TDOA location	88.3	20	235	230.31	22.24
	91.5	110	230	260.50	76.97
	89.1	350	550	515.22	137.25
	91.9	950	950	929.26	275.76
Proposed Genetic algorithm for TDOA location	88.3	40	12	13.05	6.49
	91.5	78	22	23.45	14.89
	89.1	140	56	51.57	16.87
	91.9	158	60	48.87	24.09

## Notes

**Author Contributions:** Y.-T.C. supervised the theoretical and experimental work, analyzed the characterization results and finalized the manuscript. C.-L.W. measured the experimental data. H.-C.C. developed and designed the genetic algorithm. All authors read and approved the final manuscript.

## Appendix

Based on Figure A1 published in [32], the derivation process used in locating the transmitter position (*X_T_*,*Y_T_*) is formulated as follows:

(A1) $$d_{1}=\sqrt{{\left(X_{T}-X_{1}\right)}^{2}+{\left(Y_{T}-Y_{1}\right)}^{2}}$$

(A2) $$d_{2}=\sqrt{{\left(X_{T}-X_{2}\right)}^{2}+{\left(Y_{T}-Y_{2}\right)}^{2}}$$

(A3) $$d_{3}=\sqrt{{\left(X_{T}-X_{3}\right)}^{2}+{\left(Y_{T}-Y_{3}\right)}^{2}}$$

Subtract the square of Equation (A2) from the square of Equation (A1) as follows:

(A4) $$\begin{array}{c} d_{1}^{2}-d_{2}^{2}={\left(X_{T}-X_{1}\right)}^{2}+{\left(Y_{T}-Y_{1}\right)}^{2}-{\left(X_{T}-X_{2}\right)}^{2}+{\left(Y_{T}-Y_{2}\right)}^{2}\\ =-2X_{T}X_{1}+2X_{T}X_{2}+X_{1}^{2}-X_{2}^{2}-2Y_{T}Y_{1}+2Y_{T}Y_{2}+Y_{1}^{2}-Y_{2}^{2}\\ =-2X_{T}(X_{1}-X_{2})+X_{1}^{2}-X_{2}^{2}-2Y_{T}(Y_{1}-Y_{2})+Y_{1}^{2}-Y_{2}^{2} \end{array}$$

Rearrange Equation (A4) as follows:

(A5) $$2\left(X_{1}-X_{2}\right)X_{T}+2\left(Y_{1}-Y_{2}\right)Y_{T}=X_{1}^{2}-X_{2}^{2}+Y_{1}^{2}-Y_{2}^{2}-d_{1}^{2}+d_{2}^{2}$$

Similarly, the relationship between d_1_ and d_3_:

(A6) $$2\left(X_{1}-X_{3}\right)X_{T}+2\left(Y_{1}-Y_{3}\right)Y_{T}=X_{1}^{2}-X_{3}^{2}+Y_{1}^{2}-Y_{3}^{2}\;-d_{1}^{2}+d_{3\;}^{2}$$

Combine Equations (A5) and (A6) into matrix form as follows:

(A7) $$2\text{B}\left[\begin{array}{c} X_{T}\\ Y_{T} \end{array}\right]=\text{A}\;\;\;\;\;\;or\;\;\;\;\;\;\text{A}-2\text{B}\left[\begin{array}{c} X_{T}\\ Y_{T} \end{array}\right]=0$$

where the matrix

(A8) $$\text{B}=\left[\begin{array}{cc} X_{1}-X_{2} & Y_{1}-Y_{2}\\ X_{1}-X_{3} & Y_{1}-Y_{3} \end{array}\right]$$

the matrix

(A9) $$\text{A}=\left[\begin{array}{c} X_{1}^{2}-X_{2}^{2}+Y_{1}^{2}-Y_{2}^{2}-d_{1}^{2}+d_{2}^{2}\\ X_{1}^{2}-X_{3}^{2}+Y_{1}^{2}-Y_{3}^{2}-d_{1}^{2}+d_{3}^{2} \end{array}\right]$$

To derive the range (distance) difference of the TDOA approach, the following relationship is applied:

(A10) $$d_{21}=c\cdot t_{21}=ct_{2}-ct_{1}=d_{2}-d_{1}$$

(A11) $$d_{31}=c\cdot t_{31}=ct_{3}-ct_{1}=d_{3}-d_{1}$$

where *d_ij_* denotes the distance between the *i*-th and *j*-th stations. The symbol *c* denotes the radio wave speed of 3 × 10^8^ m/s.

Rearrange Equations (A10) and (A11) as follows:

(A12) $$\;\;d_{2}=d_{1}+d_{21}$$

(A13) $$d_{3}=d_{1}+d_{31}$$

The square of Equations (A12) and (A13) is obtained as follows:

(A14) $$d_{2}^{2}=d_{1}^{2}+2d_{1}d_{21}+d_{21}^{2}$$

(A15) $$d_{3}^{2}=d_{1}^{2}+2d_{1}d_{31}+d_{31}^{2}$$

Equations (A14) and (A15) are rearranged as follows:

(A16) $$-d_{1}^{2}+d_{2}^{2}=2d_{1}d_{21}+d_{21}^{2}$$

(A17) $$-d_{1}^{2}+d_{3}^{2}=2d_{1}d_{31}+d_{31}^{2}$$

Substitute the matrix **A** into Equations (A16) and (A17):

(A18) $$\text{A}=\left[\begin{array}{c} X_{1}^{2}-X_{2}^{2}+Y_{1}^{2}+2d_{1}d_{21}+d_{21}^{2}\\ X_{1}^{2}-X_{3}^{2}+Y_{1}^{2}+2d_{1}d_{31}+d_{31}^{2} \end{array}\right]$$

Substitute Equation (A7) described as below into **A** and **B** matrix of Equations (A18) and (A8), respectively:

$$2\text{B}\left[\begin{array}{c} X_{T}\\ Y_{T} \end{array}\right]=\text{A}\;\;\;\;\;\;or\;\;\;\;\;\;\text{A}-2\text{B}\left[\begin{array}{c} X_{T}\\ Y_{T} \end{array}\right]=0$$

The interference station (longitude, latitude) of $\left[\begin{array}{c} X_{T}\\ Y_{T} \end{array}\right]$ is thus solved.
